# Median nerve block increases the success rate of radial artery cannulation in women with gestational hypertension undergoing cesarean section

**DOI:** 10.1186/s12871-022-01793-4

**Published:** 2022-08-05

**Authors:** Xin Men, Qian Wang, Wen-shen Hu, Yun Chai, Ting-ting Ni, Hong-ye Sho, Zhen-feng Zhou

**Affiliations:** 1 Department of Anesthesiology, Hangzhou Women’s Hospital (Hangzhou Maternity and Child Health Care Hospital, Hangzhou First People’s Hospital Qianjiang New City Campus, The Affiliated Women’s Hospital of Hangzhou Normal University), Hangzhou, 315014 China; 2 Department of Anesthesiology, The Affiliated ZheJiang Hospital, School of Medicine, Zhejiang University, Hangzhou, 315014 China; 3 Department of Obstetrics, Hangzhou Women’s Hospital (Hangzhou Maternity and Child Health Care Hospital, Hangzhou First People’s Hospital Qianjiang New City Campus, The Affiliated Women’s Hospital of Hangzhou Normal University), Hangzhou, 315014 China; 4 Department of Anesthesiology, Ningbo NO.7 Hospital, Ningbo, 320000 China

**Keywords:** Nerve block, Radial artery puncture, Pregnancy, Hypertension

## Abstract

**Background:**

The radial artery cannulation helps to maintain the stability of maternal hemodynamics and reduce complications, however, it is difficult for women with gestational hypertension. Ultrasound-guided median nerve block can cause arterial vasodilation, which may improve the success rate of radial artery cannulation.

**Methods:**

Ninety-two women with gestational hypertension and risks of intra-operative bleeding undergoing cesarean section following failed ultrasound-guided cannulation were identified and randomized into the median nerve block group and control group. Median nerve block was performed under the guidance of ultrasound in the middle forearm and 5 ml of 0.5% lidocaine was injected. Subcutaneous local block was administered in the control group. The ultrasound-guided radial artery cannulation was performed ten minutes after blocking. Baseline measurements (T1) were performed after 10 minutes of rest. All variables were measured again at 10 (T2) and 30 (T3) minutes after median nerve block or local block. The primary outcome was the success rate of radial artery cannulation within 10 minutes after blocking. The puncture time, number of attempts, the overall complications, and ultrasonographic measurements including radial artery diameter and cross-sectional area were recorded before (T1), 10 minutes (T2) after, and 30 minutes (T3) after block.

**Results:**

A total of 92 pregnant women were identified and completed the follow-up. As compared to control group, the first-attempt success rate of radial artery cannulation was significantly higher (95.7% vs78.3%, *p* = 0.027) and procedure time to success was significantly shorter (118 ± 19 s vs 172 ± 66 s, *p* < 0.001) in median nerve group. Median nerve group also had a significantly less overall number of attempts (*p* = 0.024). Compared with control group, the diameter and cross-sectional area of radial artery increased significantly at the T2 and T3 points in median nerve group (*p* < 0.001), as well as percentage change of radial artery diameter and CSA. No difference was observed in the overall complication at chosen radial artery, which including vasospasm (21.7% vs 28.3%; *p* = 0.470) and hematoma (4.3% vs 8.7%; *p* = 0.677).

**Conclusions:**

Ultrasound-guided median nerve block can increase the first-attempt success rate of chosen radial artery cannulation in women with gestational hypertension and risks of intra-operative bleeding undergoing cesarean section following failed radial artery cannulation, and especially for those anesthesiologists with less experienced in radial artery cannulation.

**Trial registration:**

ChiCTR2100052862; http://www.chictr.org.cn, Principal investigator: MEN, Date of registration: 06/11/2021.

**Supplementary Information:**

The online version contains supplementary material available at 10.1186/s12871-022-01793-4.

## Introduction

Globally, approximately 10% of women have gestational hypertension during pregnancy, and hypertensive disease remains an important cause of maternal and neonatal morbidity and mortality [1, 2]. Gestational hypertension includes preeclampsia and severe preeclampsia. The incidence of gestational hypertension may increase with the increased incidence of obesity, maternal age, and comorbidities [1]. Women with severe preeclampsia should receive the same standards of critical care as other acutely unwell patients [1]. Radial artery cannulation is a frequent procedure in emergency and critical care that helps to maintain the stability of maternal hemodynamics and reduce complications. Radial artery cannulation has several advantages, including shallow location, easy compression, distance from important nerves, presence of a collateral supply network, low complication rate, and unrestricted patient activity [3, 4].

However, it is difficult for women with gestational hypertension, as the basic pathological feature of gestational hypertension is systemic arteriole spasm [1]. Ultrasound guidance can significantly improve the success rate of radial artery cannulation [5–7]; however, the success rate of initial cannulation still varies from 51 to 95%. Furthermore, the success rate of initial cannulation in patients with gestational hypertension is unclear [8–12].

Currently, many studies have aimed to improve the success rate of arterial cannulation by expanding the arterial lumen. A recent study showed that median nerve block increased radial artery flow velocity and vascular dilation to a greater extent than other forearm nerve blocks [13, 14]. However, compared with traditional subcutaneous local anesthesia, it remains unclear whether forearm nerve block can improve the success rate of radial artery cannulation after the first attempt fails in women with gestational hypertension. Therefore, this study evaluated the effect of ultrasound-guided median nerve block on the diameter, blood flow, and area of the radial artery in women with gestational hypertension, as well as the success rate of radial artery puncture.

## Methods

### Study design

This study was approved by The Ethics Committee of Hangzhou Women’s Hospital (IRB:2021-A(6)-19). This prospective, randomized, and double-blind trial was conducted from November 10, 2021, to April 5, 2021, after receiving written informed consent from all participants, and 92 women were recruited.

Women with gestational hypertension and risks of intra-operative bleeding who should receive intraoperative invasive arterial blood pressure monitoring undergoing elective cesarean section will be screened, and the first attempt of radial artery puncture with ultrasound guidance will fail. Severe preeclampsia was defined as a BP of 160/100 mmHg or greater on two occasions at least 4 h apart while the patient was on bed rest. Risks of intra-operative bleeding were placental abruption, placental implantation, multiple cesarean section and other risks according to the obstetrician’s decision.

The exclusion criteria were emergency surgery, modified Allen’s test negative (Modified Allen’s test: In this modified test, the clinician compresses both the ulnar and radial arteries of the hand, while the patient repeatedly clenches the hand into a fist, causing the hand to blanch. The test is considered normal if the palm reddens less than ten seconds after release of pressure over the ulnar artery.), infection or external injury at or near the puncture site, history of coagulopathy, and history of vascular diseases such as vasculitis.

Standard monitoring was applied, and we suggested applying intraoperative invasive arterial blood pressure monitoring for women with gestational hypertension and risks of intra-operative bleeding during cesarean section at our medical institution. All women were supine with the arm extended to 60° and supported on the arm board during radial artery cannulation and ultrasonographic measurements.

### Randomization and blinding

The pregnant women were randomly divided into a median nerve group and a control group at a 1:1 ratio by an independent researcher using numbered sealed envelopes. An independent researcher performed median nerve block. a doctor experienced in vascular ultrasound examination performed the ultrasound examinations, who was blind to the group allocation. The outcome assessor who was unaware of the group allocation evaluated the cross-sectional area and diameter of the radial artery of all patients according to the ultrasound images. In addition, the included patients, surgical doctors, and data analysts were all blind to the group assignments.

### Median nerve block

**Fig. 1:**
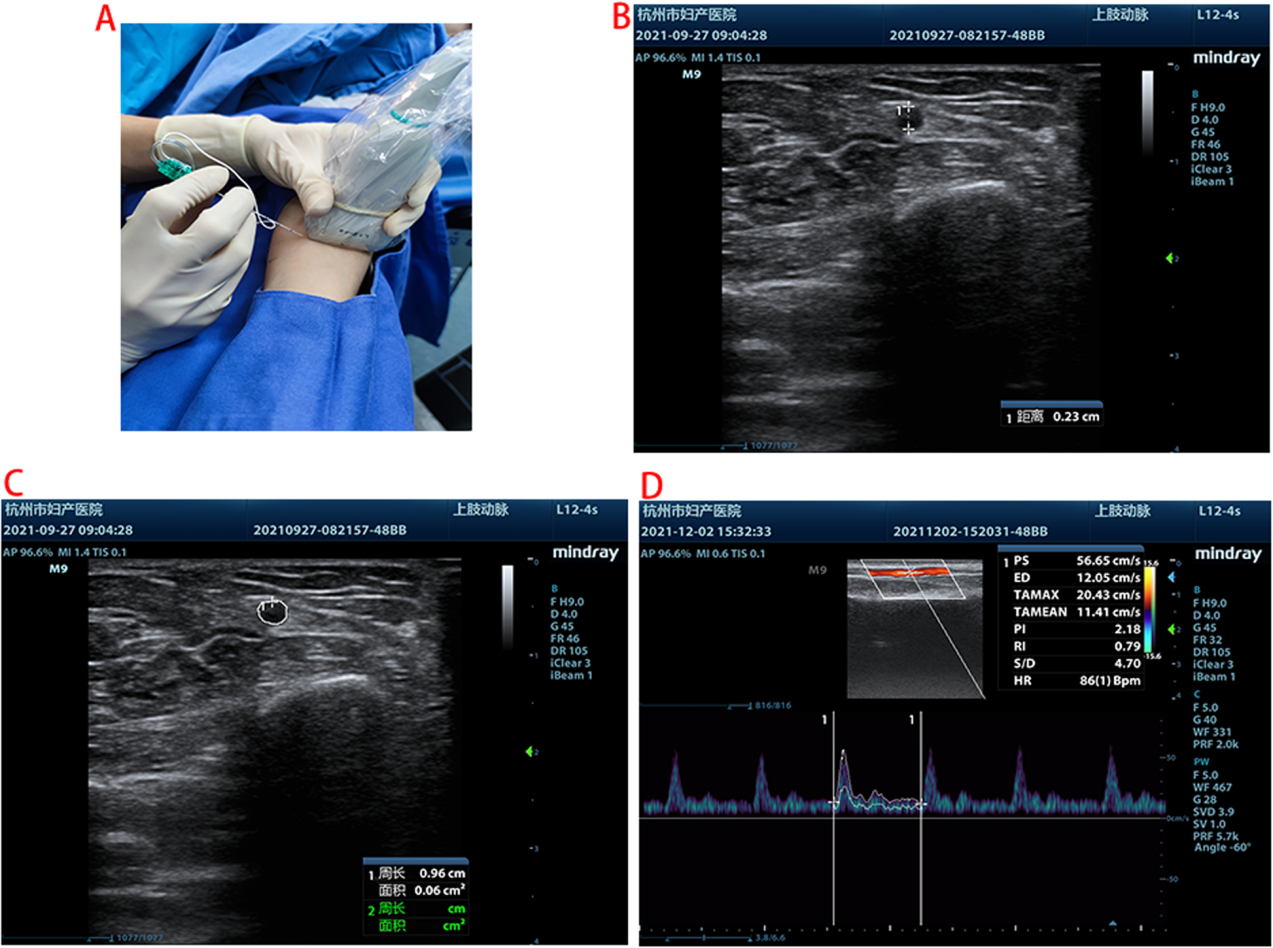
Median nerve block and ultrasonographic measurements

The forearm was placed in the supine position. A sterile gel was applied to provide ultrasonic coupling between the skin surface and the transducer. Under all aseptic precautions, median nerve block was performed under the guidance of ultrasound in the middle forearm [13] using 22G Stimuplex D nerve needles (Fig. 1A), and 5 ml of 0.5% lidocaine was injected. The location of the nerve block was the middle forearm (between shallow and deep compartments) [13]. Subcutaneous local block was administered in the control group without nerve block.

According to the preliminary test, compared with 1% lidocaine, we found that 0.5% lidocaine in 5 ml of median nerve block could dilate the radial artery to the same extent but had less effect on the movement and sensory function of the finger. The local anesthetic dose for median nerve block was determined based on the dose per nerve block for adult forearm nerve block. In adult hand and wrist surgery, 4 ~ 5 ml local anesthetic for each nerve can meet the surgical needs [14].

### Ultrasonographic measurements

Baseline measurements (T1) were performed after 10 minutes of rest. All variables were measured again at 10 (T2) and 30 (T3) minutes after median nerve block or local block.

The ultrasonic probe was placed longitudinally over the radial artery, and ultrasonographic measurements were recorded close to 2 cm from the radial styloid process. A Mindray machine (M9) with a 4–12 MHz variable frequency linear array transducer and a 1-5 MHz variable frequency convex array ultrasound transducer was applied. A set of vital signs and ultrasonographic parameters, such as a short-axis (out-of-plane) view of radial artery internal diameter (Fig. 1B) and the cross sectional area (Fig. 1C), were measured [5, 15]. Radial artery diameter is measured by an electronic caliper on a machine as the vertical distance between the inner walls of an artery [14], and the image corresponding to the end diastole is located by freezing the image at the end diastole.

After activating pulse Doppler ultrasound (PWD) mode, the volumetric gate was placed in the center of the arterial lumen. The arterial hemodynamics parameter package was displayed. The ultrasound angle was defined as the angle between the direction of blood flow and the ultrasound beam and remained between 30° and 60° in the process of the examination. The time average maximal velocity (TAMAX) was recorded as shown in Fig. 1D. According to a previous study [13], the volume of blood flow was calculated as follows: volume of blood flow (ml/sec) = cross-sectional area (CSA, cm2) × TAMAX (cm/sec).

### Radial artery Cannulation with ultrasound guidance

The radial artery was used 10 minutes after the failed first attempt of radial artery puncture with ultrasound guidance. We performed modified Allen’s test to ensure that the ulnar artery was unobstructed. The puncture point was close to 2 cm from the radial styloid process. The operator performed ultrasound-guided puncture 10 minutes after blocking, and the puncture time was recorded. Invasive arterial blood pressure was monitored after radial artery cannulation.

We applied the short-axis view (out-plane) technique and a 20-gauge arterial catheter to perform arterial cannulation [16, 17]. During ultrasound-guided arterial catheterization, the operator was not allowed to intentionally puncture both walls of the artery (transfixion technique). However, to eliminate the influence of other techniques on the success rate and operation time, guide wires were not allowed in the study population. When the arterial waveform was confirmed on the monitor, it was assumed that the cannulation had been completed. If cannulation was not accomplished in 10 minutes, the case was considered to have failed at the selected radial artery [18]. The total operation time of arterial cannulation was defined as the time interval from the first puncture of the skin with the needle catheter to the confirmation of the arterial waveform on the monitor, regardless of the position of the arterial cannula. We also recorded intraoperative complications, including hematoma and vasospasm, which were defined as the radial artery diameter decreasing by more than 25% after cannulation [4, 19–24]. The patients’ characteristics was collected, including age, weight, and American Society of Anesthesiologists physical condition classification.

### Primary outcome

The primary outcome was the success rate of radial artery cannulation within 10 minutes after blocking. Secondary outcomes included the puncture time, number of attempts, radial artery diameter, and cross-sectional area before, 10 minutes after, and 30 minutes after block and the overall complications. According to previous studies, the duration of tibial nerve and common peroneal nerve sensory block was assessed by cold stimulation and bilateral comparison [25]. Thus, the nerve block duration was defined as the amount of time the two fingers felt similar.

### Sample size

The success rate of radial artery puncture was 0.53 with ultrasound guidance [12], and the median nerve block can increase the cross-sectional area (nearly 3-fold) of the radial artery [26]. We assumed that the success rate would be improved with median nerve block by at least 50% in women with gestational hypertension. A one-tailed chi-squared test was performed, and we estimated that 88 patients were required to provide 80% power with a type I error probability of 0.05. Assuming that the follow-up loss rate was 5%, a total of 92 cases were needed. Analysis was computed using G-Power (version 3.1; Informer Technologies, Inc.).

### Statistical analysis

Data analysis was performed with SPSS V.18.0 (SPSS, Chicago, Illinois, USA). Normally distributed variables will be expressed as the mean ± SD plus 95% confidence interval (95% CI), and categorical variables will be expressed as frequency (percentage age). The Pearson test was used for the correlation analysis. Nonnormally distributed data are shown as the medians and interquartile ranges. Analysis of variance for repeated measurements was applied for repeated measured variables between the groups and the different times. Differences between the groups measured at the same time points were analyzed using Student’s two-sample t test or the Mann–Whitney U test. Two-sided *p* values are shown, and the limit of statistical significance was set to *p* < 0.05.

## Results

### Baseline clinical parameters

**Fig. 2:**
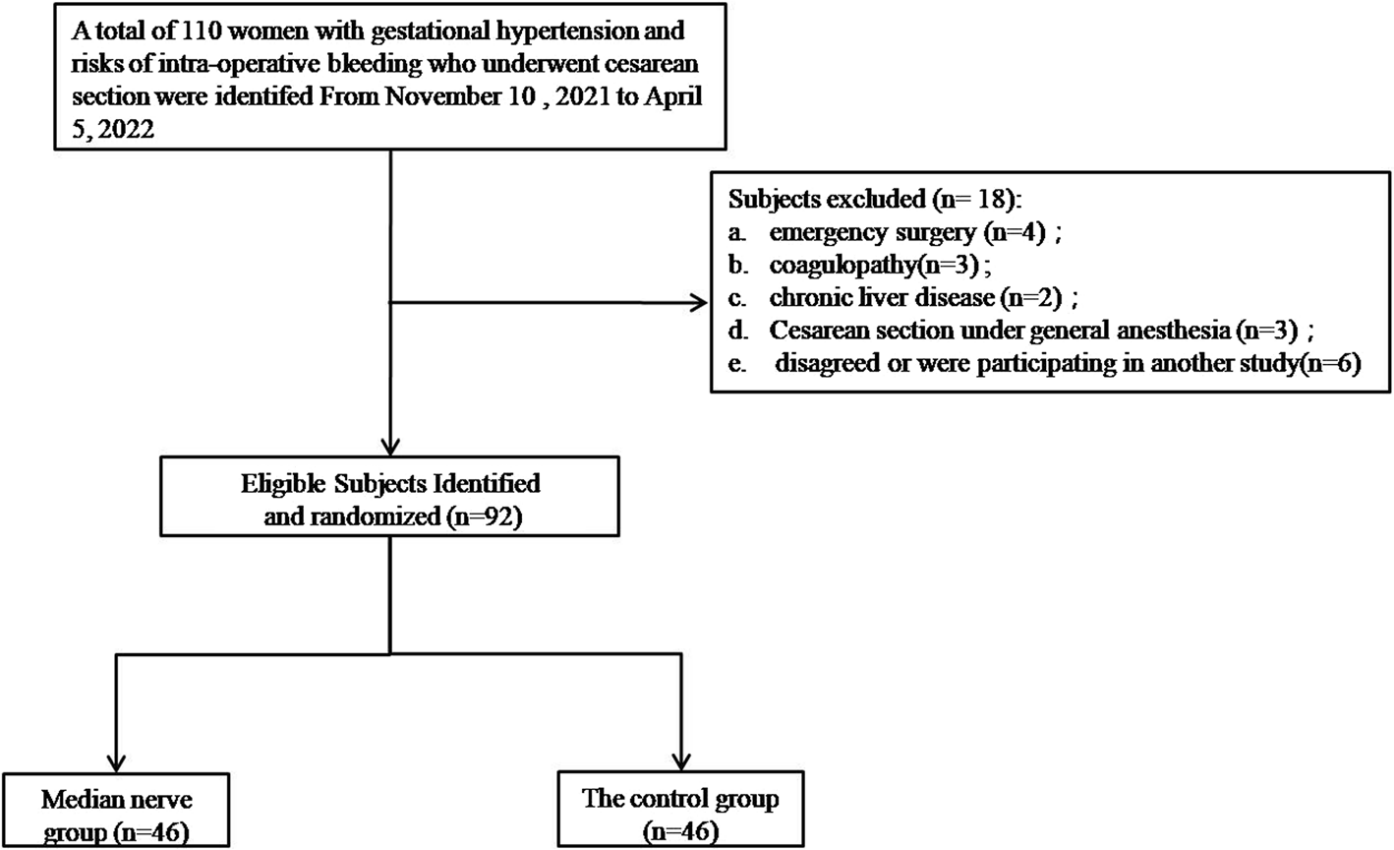
Study flowchart Study population recruitment summary

Of the 110 women with gestational hypertension and risks of intra-operative bleeding who underwent cesarean section, 92 patients were identified and completed the follow-up, and 18 patients met the exclusion criteria, as shown in Fig. 2. The basic parameters of the two groups are summarized in Table 1, and no difference was found.

**Table 1:** Patient characteristics of control group and median nerve group for radial artery cannulation

	Control (*N* = 46)	Median nerve (*N* = 46)	*p*-value
Age(y)	32 ± 5	32 ± 4	0.602
Estimated gestational age (wk)	37.4 ± 1.8	37.5 ± 1.6	0.618
Body mass index (kg/m^2^)	27 ± 4	27 ± 3	0.933
Baseline systolic pressure (mm Hg)	137 ± 15	137 ± 15	0.955
Baseline diastolic pressure (mm Hg)	80 ± 13	79 ± 10	0.593
Baseline heart rate (bpm)	84 ± 15	84 ± 15	0.961
Preeclampsia			> 0.999
Mild	31(67.4%)	32(69.6%)	
Severe	15(32.6%)	14(30.4%)	
Antihypertension treatment			0.770
Labetolol	14(30.4%)	13(28.3%)	
Nicardipine	3(6.5%)	5(10.9%)	
Nifedipine	0(0%)	0(0%)	
Magnesium	4(8.7%)	6(13.0%)	

Values are mean ± SD or number (proportion)

The average age of the study population was 32 years, the average BMI (Body mass index) was 27, the average estimated gestational age was 37 week, 31.5% had severe preeclampsia, and the other patients had mild preeclampsia.

Over time, no significant differences were observed in blood pressure and HR between the two groups.

### Radial artery Cannulation

Compared to the control group, the first-attempt success rate of radial artery cannulation was significantly higher (95.7% vs. 78.3%, *p* = 0.027), and the procedure time to success within the first attempt was significantly shorter (118 ± 19 s vs. 172 ± 66 s, *p* < 0.001) in the median nerve group. The median nerve group also had a significantly lower overall number of attempts (*p* = 0.024). However, there was no difference in the second-attempt success rate within 10 min (95.7% vs. 91.3%; *p* = 0.677) between the two groups. No difference was observed in the overall complications at the chosen radial artery, including vasospasm (21.7% vs. 28.3%; *p* = 0.470) and hematoma (4.3% vs. 8.7%; p = 0.677) (Table 2). The mean duration of sensory block time was 70.2 mins in the median nerve group, and no other complications were observed.

**Table 2:** Results of radial artery cannulation in control group and median nerve group

Variables	Control (*N* = 46)	Median nerve (*N* = 46)	*P* value
First-attempt success rate ofradial artery cannulation	36/46(78.3%)	44/46(95.7%)	0.027#
Procedure time to success within the firstattempt (s)	172 ± 66	118 ± 19	< 0.001
Second-attempt success rate within 10 min(%)	42/46(91.3%)	44/46(95.7%)	0.677#
Overall number of attempts (1/2/3 attempt, n)	36/6/4	44/0/2	0.024
Overall complication at first chosen radial artery			
Vasospasm	13/46(28.3%)	10/46(21.7%)	0.470
Hematoma	4/46(8.7%)	2/46(4.3%)	0.677#

Values are mean ± SD, or number (proportion)

### Ultrasonographic measurements

**Fig. 3:**
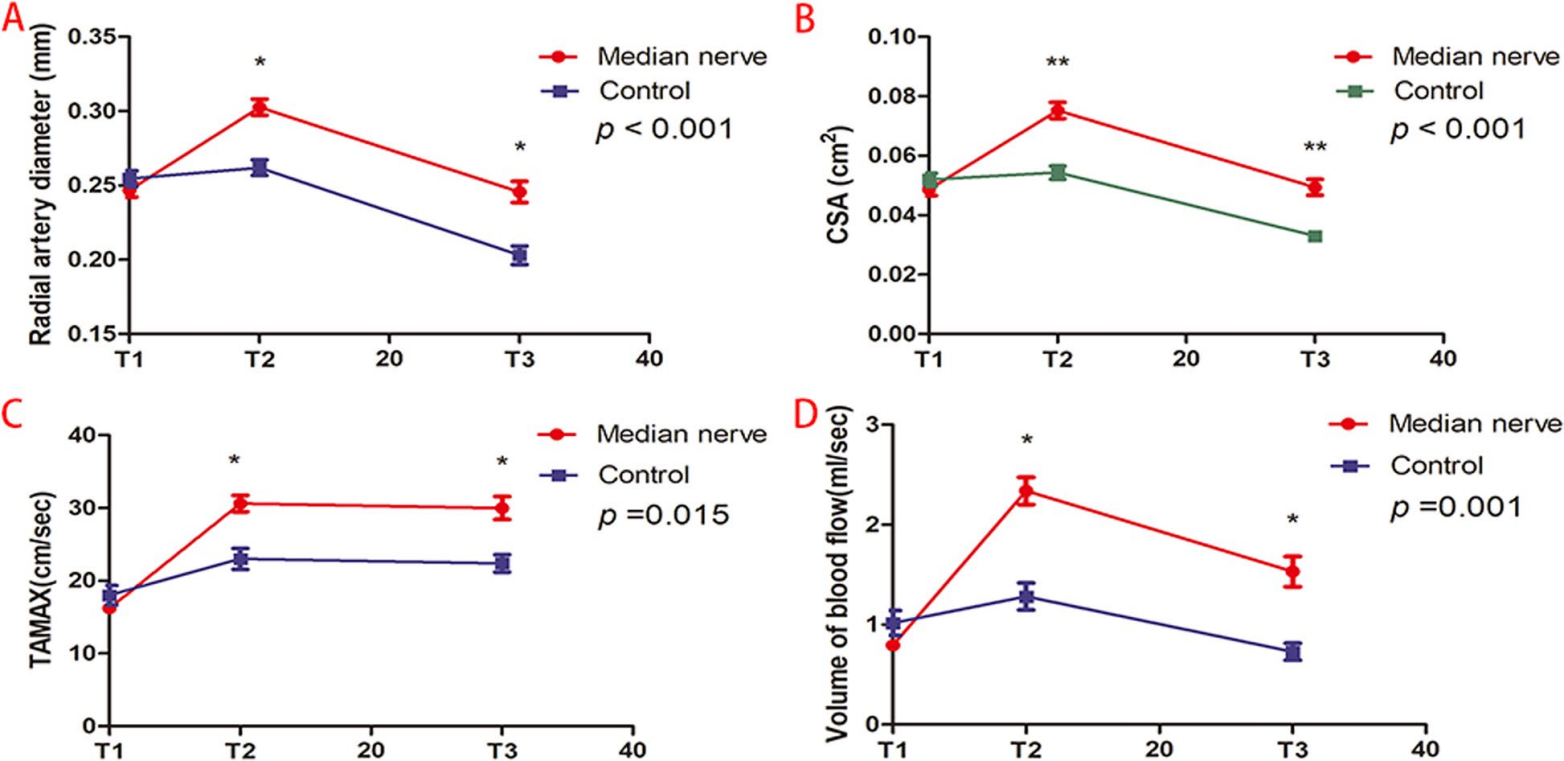
CSA = Cross-sectional area; TAMAX = Time Average Maximal Velocity. T1 = Baseline; T2 = 10 minutes after median nerve block or local anesthesia; T3 = 30 minutes after median nerve block or local anesthesia

Over time, the diameter and CSA of the radial artery showed significant differences between the two groups (*p* < 0.001). Compared with the control group, the diameter and cross-sectional area of the radial artery increased significantly at the T2 and T3 points in the median nerve group(*p* < 0.001), as well as the percentage change in radial artery diameter and CSA (Table 3, Fig. 3A for diameter and Fig. 3B for CSA).

**Table 3:** Results of ultrasonographic measurements in control group and median nerve group

	Control (*N* = 47)	Median nerve (*N* = 47)	*P* value
Radial artery diameter (mm)			< 0.001
T1	0.25 ± 0.04	0.25 ± 0.03	
T2	0.26 ± 0.04	0.30 ± 0.04	
T3	0.20 ± 0.04	0.25 ± 0.05	
Percentage change of radial artery diameter at T2(%)	4(0–8)	22(14–28)	< 0.001
Percentage change of radial artery diameter at T3(%)	−17[(−24)-9]	0[(−4.9)-8.7]	< 0.001
CSA (cm^2^)			< 0.001
T1	0.052 ± 0.015	0.049 ± 0.014	
T2	0.054 ± 0.015	0.075 ± 0.018	
T3	0.033 ± 0.013	0.049 ± 0.018	
Percentage change of CSA at T2(%)	0(0–17)	50(33–67)	< 0.001
Percentage change of CSA at T3(%)	−33[(−41)-(−25)]	0[(−3.6)-25.0]	< 0.001
TAMAX (cm/sec)			0.015
T1	18 ± 9	16 ± 4	
T2	23 ± 10	31 ± 8	
T3	22 ± 8	30 ± 11	
Volume of blood flow (ml/sec)			< 0.001
T1	1.02 ± 0.84	0.79 ± 0.32	
T2	1.28 ± 0.92	2.34 ± 0.92	
T3	0.73 ± 0.57	1.53 ± 1.03	

Values are mean ± SD, median (interquartile range) [range].T1 = Baseline; T2 = 10 minutes after median nerve block or local anesthesia; T3 = 30 minutes after median nerve block or local anesthesia. *CSA* Cross-sectional area, *TAMAX* Time Average Maximal Velocity

Over time, the TAMAX (*P* = 0.015) and volume of blood flow (*P* < 0.001) also showed significant differences between the two groups. Compared with the control group, TAMAX and volume of blood flow both increased significantly at the T2 and T3 points in the median nerve group (*p* < 0.001) (Table 3, Fig. 3C for TAMAX, Fig. 3D for blood flow).

## Discussion

The main finding of this study was that ultrasound-guided median nerve block increased the first-attempt success rate of radial artery cannulation by increasing the diameter and cross-sectional area of the radial artery in women with gestational hypertension undergoing cesarean section following failed radial artery cannulation. We also found that the median nerve block decreased time to cannulation and the overall number of cannulation attempts.

Successful radial artery cannulation was challenging for pregnant women with gestational hypertension. First, the anesthesiologists have less experienced in ultrasound-guided arterial cannulation in this obstetrics and gynecology hospital. Second, the main challenge for successful radial artery catheterization in patients was the small size of the artery [18]. Generalised vasoconstriction is a feature of gestational hypertension [1]. Vasospasm or hematoma caused by failed attempts further reduces the inner diameter of the artery and the success rate of radial artery cannulation [18, 21]. Since the radial artery is dominated by α1-adrenergic receptors [27], the radial artery is prone to vasospasm during arterial cannulation attempts. Up to 57% of cases have transient vasospasm immediately after radial artery cannulation [28], and during adult transradial artery catheterization, the incidence of persistent vasospasm is 4 to 20% [19]. After the occurrence of vasospasm, the radial artery was successfully cannulated in nine cases and failed in five of the 14 patients in the control group. As a result, the control group had a higher risk of vasospasm or complete occlusion. Radial artery catheterization was successful in eight cases and failed in two cases in the median nerve group. The relatively large diameter of the radial artery in the median nerve block group may have increased the success rate of the second attempt. Additionally, a previous study noticed that the depth of the radial artery affects ultrasound-guided cannulation [28]. Compared to local block, a relatively lower depth of median nerve block might also be due to the higher success rate, although we did not measure depth. Furthermore, a subcutaneous local block might inject air bubbles, which can mask ultrasound images of the radial artery, which might contribute to a low success rate.

We found that the median nerve block not only increases the diameter and CSA of the radial artery but also increases Tamax and velocity blood flow, which is consistent with previous studies [13, 14]. There were several reasons that we performed median nerve block to increase the success rate of radial artery cannulation following failed cannulation. Ting Li et al. found that the blood flow of the radial artery was only increased by median nerve block but not by blockade of radial/musculocutaneous nerves [26]. A recent study also confirmed that median nerve block alone could lead to arterial vasodilation, and no added benefit was found regarding other radial blocks along with median nerve block [13]. Finally, the median nerve block became easier and safer with the advent of ultrasound guidance.

We also noticed that the nerve block subsidence time of median nerve block was 70.2 mins, which means that women rarely return to the ward with numbness and weakness in their fingers. Therefore, it does not affect the baby being placed on the mother’s chest for skin-to-skin contact after delivery.

Although nerve blocks became easier and safer with ultrasonic guidance [7, 12] and we did not observe any other complications of median nerve block in this study, ultrasonic-guided median nerve block was still an intrusive operation, which raises potential concerns regarding complications such as nerve damage. We suggest that ultrasonic-guided median nerve block should be administered just after failed radial artery cannulation but not for routine use.

## Limitations

The current study has some limitations. First, the total sample was relatively small. Second, we only assessed the radial artery diameter and distal perfusion intraoperatively. Since the time of extubation varies with the type of surgery and clinical conditions, we cannot assess the diameter of the radial artery and the distal perfusion after extubation.

## Conclusions

In conclusion, ultrasound-guided median nerve block can increase the first-attempt success rate of chosen radial artery cannulation in women with gestational hypertension undergoing cesarean section following failed radial artery cannulation, and especially for those anesthesiologists with less experienced in radial artery cannulation.

## Supplementary Information

**Additional file 1: Supplemental Fig. 1.** Heart rate and Blood pressure.

## Data Availability

All data generated or analysed during this study are included in this published article and its related files.

## Abbreviations

ASA: American Standards Association
BMI: Body Mass Index
BP: blood pressure
CSA: Cross-sectional Area
TAMAX: Time Average Maximal Velocity
