# Correction: Median nerve block increases the success rate of radial artery cannulation in women with gestational hypertension undergoing cesarean section

**DOI:** 10.1186/s12871-022-01807-1

**Published:** 2022-08-27

**Authors:** Xin Men, Qian Wang, Wen-sheng Hu, Yun Chai, Ting-ting Ni, Hong-yan Shou, Zhen-feng Zhou

**Affiliations:** 1 Department of Anesthesiology, Hangzhou Women’s Hospital (Hangzhou Maternity and Child Health Care Hospital, Hangzhou First People’s Hospital Qianjiang New City Campus, The Affiliated Women’s Hospital of Hangzhou Normal University), Hangzhou, 315014 China; 2 Department of Anesthesiology, The Affiliated ZheJiang Hospital, School of Medicine, Zhejiang University, Hangzhou, 315014 China; 3 Department of Obstetrics, Hangzhou Women’s Hospital (Hangzhou Maternity and Child Health Care Hospital, Hangzhou First People’s Hospital Qianjiang New City Campus, The Affiliated Women’s Hospital of Hangzhou Normal University), Hangzhou, 315014 China; 4 Department of Anesthesiology, Ningbo NO.7 Hospital, Ningbo, 320000 China

**Correction: BMC Anesthesiol 22, 248 (2022)**

**https://doi.org/10.1186/s12871-022-01793-4**

Following publication of the original article [1], the authors identified an error in the author names of Wen-sheng Hu and Hong-yan Shou.

The incorrect author names are: Wen-shen Hu and Hong-ye Sho

The correct author names are: Wen-sheng Hu and Hong-yan Shou

The author group has been updated above and the original article [1] has been corrected.
