# Supplementary material for: Median nerve block increases the success rate of radial artery cannulation in women with gestational hypertension undergoing cesarean section
Source: BMC Anesthesiol. 2022 Aug 5;22:248. doi: 10.1186/s12871-022-01793-4 (PMC9354365; doi:10.1186/s12871-022-01793-4)
Supplement: Supplementary file 1 — Additional file 1: Supplemental Fig. 1. Heart rate and Blood pressure. [file 12871_2022_1793_MOESM1_ESM.pdf]

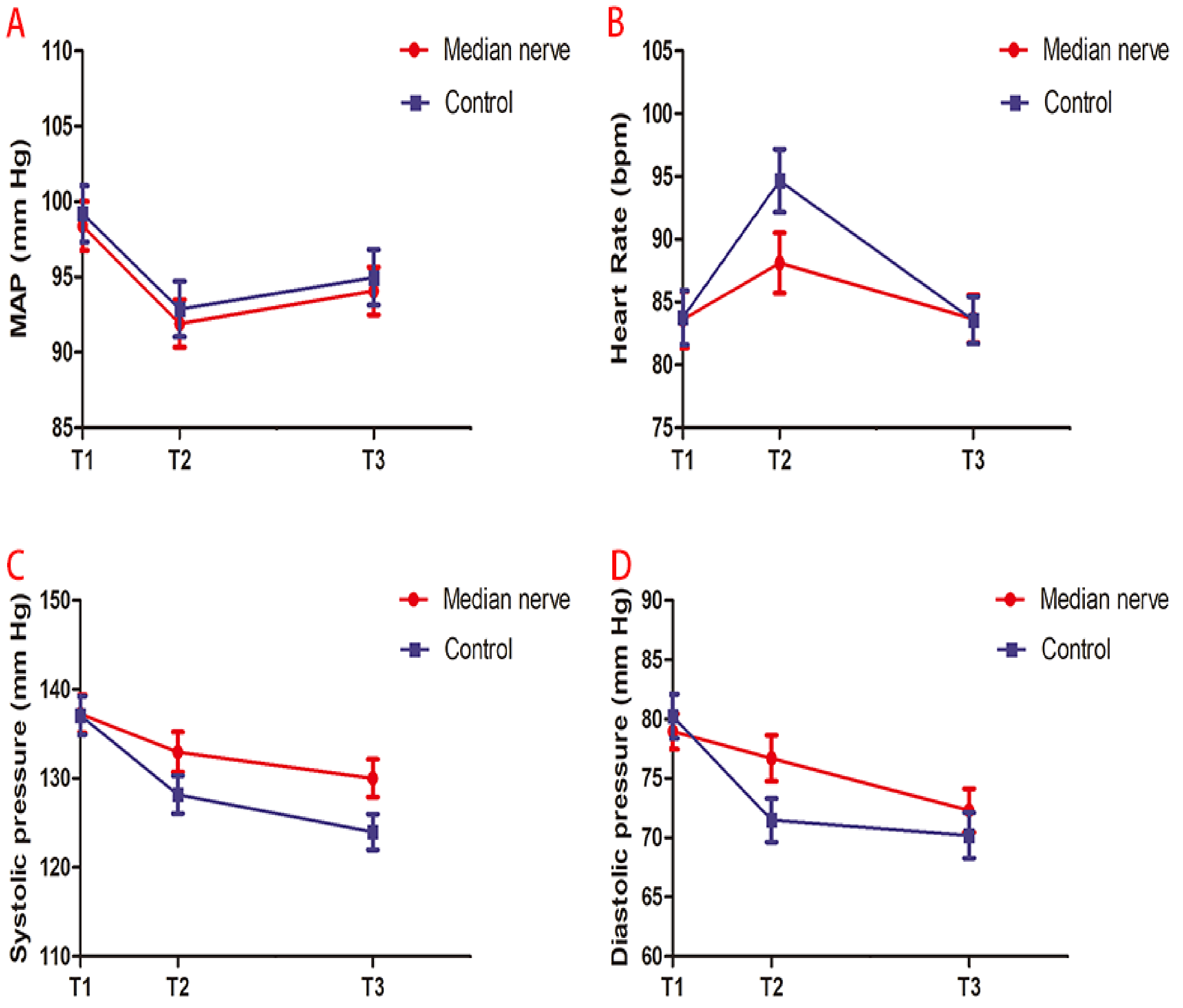

Supplemental Figure 1. Heart rate and Blood pressure. MAP= Mean arterial pressure. T1 = Baseline; T2 = 10 minutes after median nerve block or local anesthesia; T3 = 30 minutes after median nerve block or local anesthesia.
